# Hemispheric Asymmetries in Bipolar Disorder: A Systematic Review

**DOI:** 10.3390/medicina62040792

**Published:** 2026-04-20

**Authors:** Efthymia Nestora, Elena Ioannidou, Panayiotis Patrikelis, Vasiliki Folia

**Affiliations:** Laboratory of Neuropsychology and Behavioral Neuroscience, Aristotle University of Thessaloniki, 54 124 Thessaloniki, Greece

**Keywords:** bipolar disorder, hemispheric asymmetry, brain lateralization, emotional lateralization, mood disorders, cognitive function

## Abstract

*Background and Objectives*: The joint study of cerebral asymmetries and bipolar disorder (BD) has long attracted the interest of researchers and clinicians. Nevertheless, despite the increasing awareness of hemispheric asymmetries in BD, the combined investigation of these two constructs constitutes a relatively recent area of inquiry. The main objective of the present systematic review is to systematically examine the existing literature in order to identify, integrate and critically discuss evidence of hemispheric asymmetry in BD patients in terms of brain anatomy, physiology and neuropsychological function. The initial hypotheses support the presence of atypical cerebral asymmetry and differential hemispheric activation as a function of mood states in BD. *Materials and Methods*: Following the collection and analysis of numerous research papers through several databases and search engines, specific papers were identified and screened according to specified inclusion and exclusion criteria. Research papers on the adult bipolar population were included, while papers including comorbidity with other disorders, lesions, or an underage or elderly population, as well as meta-analyses and reviews, were excluded. This paper aligns with the procedures in the Preferred Reporting Items for Systematic reviews and Meta-Analyses (PRISMA 2020) guidelines, and was assessed for risk of bias according to the Cochrane guidelines by the Newcastle–Ottawa Scale (NOS). *Results*: A total of 56 papers were identified as eligible in this review. Despite inconsistent findings across the included studies, an emerging pattern suggests the presence of atypical hemispheric asymmetry in BD, both in terms of specific brain structures and functional activity. Moreover, several studies associate depressive states with increased activation of the right hemisphere, whereas manic states appear to be linked with increased activation of the left hemisphere. *Conclusions*: These findings support the aforementioned hypotheses and partly align with the theoretical framework of emotional laterality theories. However, although certain patterns were observed, a comprehensive understanding of functional hemispheric asymmetry in BD has not yet been achieved. The presence of contradictory findings highlights the need for further extensive and systematic research to improve understanding of this topic.

## 1. Introduction

Cerebral asymmetry, also referred to as brain lateralization or hemispheric specialization, describes structural and functional differences between the two cerebral hemispheres that influence both typical and atypical neuropsychological functioning. Although bilateral symmetry is a dominant anatomical feature of the human brain, notable structural and functional asymmetries exist, reflecting hemispheric specialization across cognitive and behavioral domains, known as “brain lateralization”.

While the scientific study of cerebral asymmetry is often associated with Paul Broca’s demonstration on the left hemisphere dominance of language, early observations of hemispheric differences predate his work [1]. Subsequent clinical and experimental research established that although functions are distributed across both hemispheres, they are often lateralized to varying degrees rather than being strictly localized.

Understanding bipolar disorder (BD) from a neurobiological perspective requires careful consideration of emotional processing, as emotional dysregulation is a defining feature of the disorder. Emotion is widely regarded as a multidimensional construct encompassing cognitive and behavioral patterns shaped by subjective experience [2]. Consequently, several neuropsychological theories have been proposed to explain how emotional processes are organized in the brain. Early evidence for lateralized emotional processing comes from observations of patients with left hemisphere lesions who, despite impaired speech, retained the ability to recognize emotions [3]. The Right Hemisphere Theory proposes that the right hemisphere plays a dominant role in both the perception and expression of emotions [2,4,5,6,7]. The ‘Valence Hypothesis’ suggests that positive emotions are predominantly processed in the left hemisphere, and negative emotions rely more strongly on the right hemisphere [2,4,8,9].

Davidson later introduced the Approach–Withdrawal Hypothesis, which argues that hemispheric asymmetry reflects motivational direction rather than emotional valence [10]. According to this model, approach-related emotions are associated with the left hemisphere, whereas withdrawal-related emotions are linked to the right hemisphere. EEG studies provided empirical support for this hypothesis, although some researchers criticized its focus primarily on frontal cortical activity [11]. Most recent perspectives integrate these models by suggesting that emotional processing emerges from dynamic interactions between distributed neural networks across both hemispheres [6]. Contemporary neuroimaging research further demonstrated that emotional lateralization involves dynamic, distributed networks across both hemispheres and varies depending on emotional state, individual traits, and neuropathology, suggesting plenty of alternative interpretation models [12,13].

Clinical observations of unilateral brain damage provided important evidence for emotional lateralization. Patients with right hemisphere lesions frequently exhibit disturbances in emotional expression, prosody, pragmatic communication, and social cognition, including impairments in Theory of Mind [14,15,16]. Earlier descriptions reported that right hemisphere damage was sometimes associated with euphoric or manic-like presentations, whereas left hemisphere lesions were linked to depressive or catastrophic reactions [17,18,19,20]. Although these findings were initially interpreted within simplified hemispheric models, they contributed significantly to the development of more nuanced theories of emotional asymmetry.

Bipolar disorder is a complex and highly heritable mood disorder characterized by recurrent fluctuations between manic or hypomanic states and depressive episodes [21,22]. Brain functional abnormalities mainly involve emotion-processing circuits, including hyperactive amygdala, hypoactive hippocampus and prefrontal cortex, and altered reward-related regions like the anterior cingulate and basal ganglia [23,24,25,26]. Structural studies show reduced prefrontal and hippocampal volume and enlarged amygdala, as well as altered neuroplasticity markers like BDNF [27,28,29,30]. These differences may reflect genetically guided network, rather than isolated structure, dysfunction [26].

The present systematic review aims to address a gap in the literature via identifying, analyzing and critically evaluating existing evidence regarding hemispheric asymmetries in BD, and their potential role in its pathophysiology. By integrating structural and functional findings, this review seeks to clarify whether atypical brain lateralization represents a core neurobiological feature of BD.

Although the current topic has recently drawn scientific attention, the number of reviews that pertain to hemispheric asymmetry specifically in bipolar disorder remains limited, and therefore only a few reviews that align with this topic were taken into consideration. Most of the consistent reviews focused on more general neuropsychological data and cognitive impairments in bipolar disorder, with few connections to hemispheric asymmetry [31,32,33,34]. However, most of them, when referring to hemispheric asymmetries, supported the right hemisphere involvement in depressive states and left hemisphere activation during mania. The most relevant review that was found was conducted by Moebus et al., which emphasizes the role of hemispheric asymmetry in the pathophysiology of bipolar disorder [35]. Consequently, apart from the aforementioned reviews, the research on the connection between those two variables remains insufficient, which underlines the importance of the current study.

Based on the existing literature, we propose two primary hypotheses: (1) individuals with BD exhibit atypical structural and functional hemispheric asymmetries compared to healthy controls, and (2) manic states are associated with relative left hemisphere hyperactivation, whereas depressive states are associated with relative right hemisphere hyperactivation.

## 2. Materials and Methods

### 2.1. Literature Search Strategy

The literature search was conducted between 1 September 2024 and 31 January 2025. The databases and search engines utilized were PubMed (National Library of Medicine, Bethesda, MD, USA; https://pubmed.ncbi.nlm.nih.gov/), EBSCO (EBSCO Information Services Headquarters, Ipswich, MA, USA; https://www.ebsco.com/), Scopus (Elsevier B.V., Amsterdam, The Netherlands; https://www.scopus.com/), Cambridge Core (Cambridge University Press, Cambridge, UK; https://www.cambridge.org/core), APA Psychnet (American Psychological Association, Washington, DC, USA; https://psycnet.apa.org/), SAGE Journals (SAGE Publications Ltd., London, UK; https://journals.sagepub.com/) and ScienceDirect (Elsevier B.V., Amsterdam, The Netherlands; https://www.sciencedirect.com/).

For the literature search, keywords were combined using Boolean operators (AND/OR/NOT). The following keywords and combinations were used: “Bipolar Disorder” AND (“hemispheric asymmetry” OR “cerebral asymmetry” OR “brain asymmetry” OR “lateralization” OR “brain lateralization”), “Bipolar Disorder” AND (“structural MRI” OR “functional MRI” OR “DTI” OR “EEG”), “Bipolar Disorder” AND (“left hemisphere” OR “right hemisphere”), “mood disorders” AND “hemispheric asymmetry”, “Bipolar Disorder” AND (“neuropsychology” OR “neurobiology” OR “neuroscience”), (“bipolar affective disorder” OR “manic depression” OR “bipolar depression”) AND “brain asymmetry”, “Bipolar Disorder” AND (“memory” OR “executive” OR “attention” OR “cognitive” OR “neurocognitive” OR “neuropsychological”) AND (“dysfunction” OR “impairment” OR “deficits” OR “abnormalities”), “Bipolar Disorder” NOT (“children” OR “young adults” OR “adolescents”), “Bipolar Disorder” NOT “after injury”, “Bipolar Disorder” NOT “elders”. The bibliographic material was systematically collected at multiple stages, using the aforementioned keywords in various combinations. No automated bibliography management or screening tools were used during the search process.

In order to ensure that the selected studies were consistent with the objectives of the present systematic review, specific inclusion and exclusion criteria were applied. Studies examining BD and cerebral or hemispheric asymmetry were included. Eligibility required that participants had a previous clinical diagnosis of BD type I or type II. Studies investigating brain lateralization, as well as the functional specialization of the left and the right cerebral hemispheres, were also considered. Additionally, studies employing neuroimaging and neurophysiological methods, including Magnetic Resonance Imaging (MRI), Functional Magnetic Resonance Imaging (fMRI), Diffusion Tensor Imaging (DTI), Electroencephalography (EEG), and other neuroimaging techniques, were included. In a broader context, studies examining the relationship between affective disorders and cerebral asymmetries were reviewed. Research exploring BD in relation to neuropsychology, neurobiology, and neuroscience was also considered.

Although no formal protocol was preregistered or published prior to conducting this review, it was conducted retrospectively and strictly adhered to PRISMA guidelines to ensure methodological rigor in study selection, data extraction, and statistical analyses. Recognizing the importance of preregistration in enhancing transparency and reproducibility, we are committed to registering protocols for future systematic reviews in accordance with best practices for transparency and reproducibility.

### 2.2. Selection Process

The process of identification, screening, and eligibility checking was conducted following the PRISMA guidelines. Initially, 374 studies were identified by the authors through searching the databases and search engines mentioned before. No records were identified through other types of sources. After removing duplicate records that were detected (*n* = 94), the number of studies available for screening was 280. During the screening process, the papers were evaluated based on their abstract, title and relevance to the topic of the present review. This process resulted in the exclusion of 115 records. In addition, 10 reports could not be retrieved and were therefore excluded prior to the eligibility assessment. The remaining 155 papers underwent an eligibility evaluation based on particular inclusion and exclusion criteria. A total of 109 full-text articles were excluded, as they were found to be irrelevant to the aims, objectives, and inclusion criteria proposed. The remaining 56 papers fulfilled the inclusion criteria and were therefore included in the final review.

Two of the authors (EN and EI) independently conducted the initial data extraction. Subsequently, they proceeded to cross-check the findings extracted by each party in order to ensure accuracy and sufficiency. Discrepancies were resolved through discussion and consensus, and this approach was applied uniformly across studies to maintain data integrity. The finalized data collection table provides a structured synthesis of the study characteristics and key findings. All extracted variables were evaluated for risk of bias following established guidelines, and any uncertainties regarding the quality of individual studies were reported in accordance with the criteria of the assessment tool employed (Newcastle–Ottawa Scale).

### 2.3. Inclusion Criteria

Studies were included if they met the following criteria: (1) examined individuals with BD, particularly focusing on BD and cognitive functions commonly affected by BD, such as memory, attention, and executive functioning, in order to provide a more comprehensive understanding of the disorder; (2) investigated functional impairments or dysfunctions associated with hemispheric specialization to further clarify the neurocognitive profile of individuals with bipolar disorder; (3) involved adult participants (≥18 years old); (4) included samples in which bipolar disorder was the primary diagnosis, while studies involving common psychiatric comorbidities were considered when BD-related cognitive or functional outcomes could be clearly identified; (5) employed neuropsychological, behavioral, or neuroimaging methods to assess these domains. 

### 2.4. Exclusion Criteria

During the screening process, several studies were excluded based on predefined criteria. Specifically: (1) studies involving participants with psychiatric or neurological comorbidities were excluded; (2) studies in which bipolar disorder was described as secondary to another medical or neurological condition were excluded; (3) studies primarily focusing on genetic and epigenetic factors or neurochemical alterations were excluded, as these topics were beyond the scope of the present review; (4) studies involving children, adolescents, or young adults were excluded, since the present systematic review focused exclusively on adult populations; (5) studies including elderly participants (over 75 years of age) were excluded because late-life bipolar disorder symptoms may be associated with neurodegenerative processes rather than primary bipolar disorder; (6) systematic reviews, meta-analyses, and case studies were excluded in order to focus exclusively on original empirical research relevant to the objectives of this review.

### 2.5. Risk of Bias

The papers were assessed for risk of bias, according to the Cochrane guidelines [36]. Individual studies included in the review were assessed by the Newcastle–Ottawa Scale (NOS) [37]. The score was interpreted based on the following categories: very high risk of bias, high risk of bias, and low risk of bias. Two of the authors conducted the risk of bias assessment (EN and EI), and most of the papers assessed by the authors could be classified as being at low risk of bias (a detailed RoB assessment is provided in Table S1).

## 3. Results

**Figure 1:**
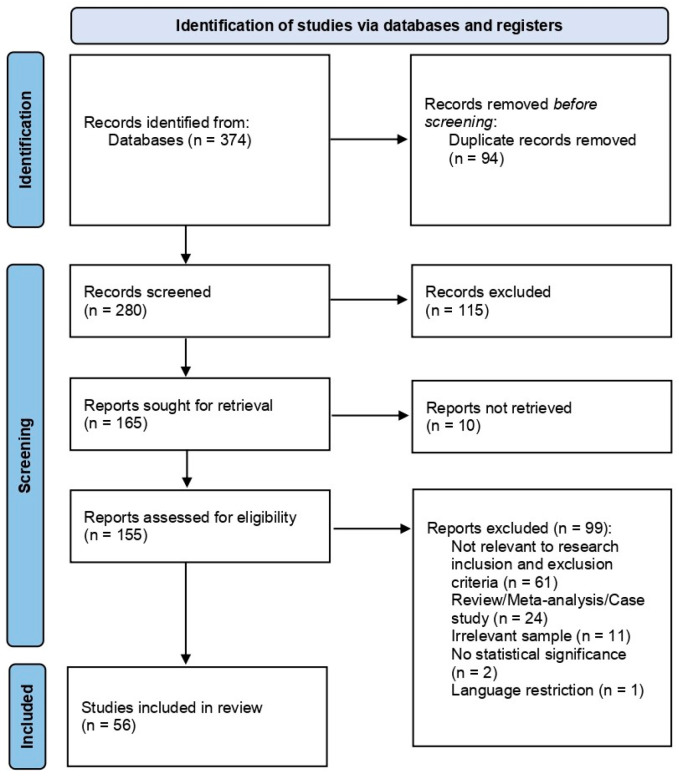
PRISMA flow diagram.

The study selection process followed the PRISMA guidelines [38]. A total of 374 studies were initially identified through database searches. After applying the inclusion and exclusion criteria, 56 studies were retained and included in the final review. The selection process is illustrated in the PRISMA flow diagram (Figure 1). A total of 56 studies were included in the present systematic review, spanning the period from 1987 to 2024, all published in English. As previously described, these studies were selected following a thorough eligibility screening process to ensure that their content aligned with the aims of this review. Some studies that followed the topic of interest had to be excluded as they did not meet the inclusion criteria. Specifically, several systematic reviews that were theoretically eligible due to their relevance to the topic were excluded [33,35]. After detailed analysis, interpretation, and evaluation of the included material, several findings were identified, grouped, and organized according to their reported outcomes.

### 3.1. Categorization of Results

To facilitate interpretation of the evidence, the extracted results were categorized based on methodology and type of evidence, resulting in three main groups: structural neuroimaging findings, functional neuroimaging findings, and Functional findings from neuropsychological assessment. The key results from all studies are summarized in Table 1.

#### 3.1.1. Structural Neuroimaging Findings

Many studies focused on anatomical and morphological asymmetries in the brains of individuals with BD. A total of 23 studies reported structural neuroimaging findings. Most of these studies used MRI (*n* = 20), while three employed diffusion tensor imaging (DTI) to assess white matter connectivity. Additionally, one study used CT scanning, and one utilized 3D laser imaging. Four studies combined multiple methodological approaches. For example, Yamada et al. used both MRI and DTI, while Kieseppä et al. combined MRI with diagnostic interviews in twins [58,91]. Likewise, Dewan et al. and Coffman et al. integrated neuropsychological tests (e.g., HRB, WAIS, and WCST) with CT or MRI data [48,49].

Findings from morphological neuroimaging studies investigating white matter asymmetry in BD are mixed. Six studies [28,52,58,60,64,70] reported heterogeneous results. Some studies identified reduced white matter volume in bipolar patients [52,58], particularly in the left hemisphere or parietal regions, while others found no significant differences [28,64] or even increased volume in specific regions, such as the posterior cingulate cortex [70]. DTI studies also revealed disrupted connectivity within networks involved in emotional regulation and executive control, specifically the default mode network and executive control network, further highlighting the heterogeneity of white matter findings in BD [60].

Several studies have also examined ventricular size in BD. Five studies reported findings primarily related to the lateral ventricles [30,58,64,65,73]. Most studies observed ventricular enlargement in bipolar patients; Strakowski et al. reported greater enlargement in the left hemisphere, whereas Manelis et al. found that longer illness duration and more severe depressive symptoms were associated with greater enlargement of the lateral ventricles [30,65]. Ventricular enlargement was also associated with altered myelin content in the insular cortex, suggesting that ventricular abnormalities may reflect broader structural changes in BD [65].

Beyond white matter alterations, several studies investigated gray matter volume [28,52,58,64,70,75]. Most studies found that significant gray matter was decreased compared with healthy controls. More specifically, overall gray matter reduction was observed, either with or without hemispheric asymmetry, while Moorhead et al. linked temporal lobe volume loss to cognitive impairment and mood instability [28,52,64].

The examination of cortical structural alterations in BD was a primary objective in several studies (*n* = 6) [39,48,64,73,76,91]. The findings were again heterogeneous. While Maller et al. reported no overall cortical volume differences, widespread, even microstructural, cortical abnormalities, particularly in frontal, dorsolateral, temporal and occipital regions, were identified.

Only a limited number of studies examined subcortical structures in BD [30,73,81]. Pearlson et al. found reduced left amygdala volume in bipolar patients, while Strakowski et al. reported enlarged amygdala and possible enlargement of thalamic and pallidal structures [30,73]. Schindler et al. observed increased hypothalamic volume in both bipolar and major depressive disorder patients, particularly in the left hemisphere [81]. The hippocampus was investigated in two studies (*n* = 2) [56,64]. Maller et al. found reduced bilateral hippocampal volume, while Javadapour et al. reported an 8.5% increase in left hippocampal volume [56,64]. Only one study examined cerebral blood volume [40]. This study reported abnormal lateralization patterns in bipolar patients, characterized by increased blood volume in the left frontal and temporal lobes. This hyperperfusion may reflect overactivation during mood episodes, particularly mania.

DTI studies often use fractional anisotropy (FA) to assess white matter integrity and connectivity (*n* = 2). In bipolar patients, FA values were reduced in anterior corpus callosum fibers compared with healthy controls, indicating disrupted microstructural organization [55,91]. Additional DTI metrics, including radial diffusivity (RD) and mean diffusivity (MD), showed increased RD in the right hemisphere and elevated MD in several regions, including the corpus callosum, cingulum, and internal and external capsules. Bipolar patients also exhibited reduced interhemispheric FA in anterior and subcortical regions, particularly in right occipital-parietal tracts, compared with both healthy controls and individuals with schizophrenia.

The majority of studies investigating brain asymmetry in BD often use the laterality index (LI) to quantify hemispheric dominance (*n* = 10) [43,48,49,52,55,60,61,65,75,89]. Overall, most studies reported atypical asymmetry in bipolar patients compared with healthy controls [52,55,60]. Bipolar individuals frequently exhibit reduced or absent typical left hemisphere dominance, possibly related to reduced left hemisphere white matter volume, as well as altered connectivity affecting emotion regulation and visuospatial processing [52]. DTI studies also reported abnormal lateralization in anterior, subcortical, and callosal tracts, often more pronounced in the right hemisphere [55,60]. Some studies also report increased asymmetry in the premotor cortex [75], anterior cingulate, and attention-related networks, while others reported normal or reduced asymmetry in specific brain regions [43,89]. Furthermore, longer illness duration and more severe depressive symptoms may be associated with reduced hemispheric asymmetry [65]. Overall, BD appears to be associated with altered hemispheric asymmetry, though specific patterns vary across studies.

Taken together, the structural neuroimaging literature suggests that BD is associated with widespread alterations in both cortical and subcortical structures, as well as disrupted hemispheric asymmetry. However, considerable variability across studies indicates that these alterations are likely influenced by clinical factors such as illness duration, medication status, and mood state.

#### 3.1.2. Functional Neuroimaging Findings

During the results recording process, part of the collected data concerned neuroimaging findings associated with functional brain asymmetry. To identify these patterns, results derived from various neuroimaging methods were examined. Specifically, findings from eight studies employing fMRI were collected [41,47,66,72,79,80,86,90]. In addition, five studies reported findings derived from EEG [54,59,71,82,85]. Furthermore, three studies included results from MEG, while one reported data collected through PET. Finally, one study presented findings collected using event-related potentials (ERPs), a method that records the brain’s responses to external sensory, motor, or cognitive stimuli by measuring the electrical activity generated by sensory processing [45]. By examining a range of neuroimaging techniques, a more comprehensive understanding of functional cerebral asymmetry in BD was obtained.

A substantial portion of the findings concerns observations related to frontal and prefrontal cortical regions, as well as the anterior brain more broadly. Studies report increased frontal activation in response to positively valenced stimuli even during depressive states, and frontal hyperactivation during manic episodes, particularly within the left frontoparietal cortex [44,59]. Similarly, increased activation of the left orbitofrontal and prefrontal cortex during depressive states, along with reduced activation of the left orbitofrontal cortex during mania, was reported [41]. Moreover, a more severe course of the disorder has been associated with increased left-hemispheric activation [71]. Further findings examining cerebral asymmetry in BD focus on the amygdala. Although Altshuler et al. reported no significant differences in amygdala activation between bipolar and non-bipolar individuals, Najt et al. demonstrated atypical cerebral organization involving the right amygdala [41,66].

Another important aspect of the findings concerns cerebral asymmetry in BD and linguistic networks. In Padovan et al.’s study, neuroimaging findings indicated a lack of lateralization during phonemic tasks and an absence of typical left lateralization during language tasks [72]. However, Royer et al. did not support the presence of reduced brain lateralization [80]. More generally, alterations in the spatial organization of the linguistic network within the left hemisphere have been observed, accompanied by atypical hyperactivation of right hemisphere regions [79,86].

Regarding the auditory cortex, asymmetries have been observed in MEG responses to auditory stimuli (M100, M200). Wang et al. reported asymmetry in the topographic location of M100 in non-bipolar participants, whereas bipolar individuals exhibited more symmetrical patterns, consistent with findings that further reported significantly reduced M100 and M200 amplitudes in bipolar groups [87,88]. Similarly, reduced hemispheric asymmetry in the primary and auditory cortex has been suggested, as well as irregularities in temporal regions [78,79,85,87,88].

Additional findings concern the motor cortex. Caligiuri et al. reported increased cerebral asymmetry in the primary motor cortex in bipolar patients, particularly within the left hemisphere, reflecting increased cortical excitability [47]. While control participants demonstrated typical bilateral activation of the supplementary motor area, with contralateral predominance depending on the hand used during tasks, this pattern was absent in bipolar individuals. Finally, Altshuler et al. reported weaker activation of the right dorsolateral prefrontal cortex during task performance, suggesting impaired recruitment of secondary task-related regions despite the involvement of working memory processes [41].

Differences were also observed in attentional processes. Specifically, neuroimaging findings reported by Padovan et al. revealed weaker functional connectivity among regions of the dorsal attention network in individuals with BD [72].

Regarding visuospatial perception and visual field processing in bipolar patients, Bruder et al. reported an atypical asymmetry in visual regions during early stages of processing, suggesting the involvement of attentional mechanisms rather than higher-order cognitive processes [45]. Event-related potentials also revealed an electrophysiological correlate of this atypical visual field asymmetry during auditory spatial tasks.

Finally, Tas et al. reported differences in concordance and coherence patterns in BD patients compared with individuals with unipolar depression [85]. Specifically, altered alpha and theta coherence in parietal-temporal regions indicated reduced perfusion and decreased communication between brain regions, potentially leading to dysfunctional information transfer and impaired emotional regulation. Additionally, reduced coherence in frontal regions may indicate disruption of interhemispheric connectivity. Regarding theta activity, Koller-Schlaud et al. reported increased theta activity in response to happy faces compared with sad faces [59].

#### 3.1.3. Functional Findings from Neuropsychological Assessment

Structural and functional neuroimaging studies reveal hemispheric asymmetries in BD, but these findings must be complemented by behavioral evidence. Behavioral manifestations of asymmetry can be examined through neuropsychological tests assessing cognition, attention, visuospatial and auditory perception, and motor skills. These tests help determine whether hemispheric asymmetry affects cognitive function in individuals with BD compared with healthy controls. In total, 25 studies were identified: 15 using only neuropsychological tests, and 10 combining neuropsychological assessment with neuroimaging methods, thereby providing valuable insight into the functional implications of brain asymmetry in BD. 

One of the most commonly used measures of hemispheric asymmetry is the dichotic listening test, in which different auditory stimuli are simultaneously presented to each ear to assess hemispheric dominance. Typically, healthy individuals exhibit a right-ear advantage associated with left hemisphere language dominance. Bruder et al. found that manic bipolar patients performed worse than controls, particularly for left-ear stimuli, suggesting right hemisphere dysfunction and reduced left hemisphere dominance [45,46]. 

Beyond the dichotic listening test, other auditory tasks have also examined asymmetry in BD. Overall, findings from auditory tasks suggest atypical hemispheric asymmetry in BD, which appears to be influenced by mood state [51,78].

Another commonly used task for assessing hemispheric asymmetry is the line bisection task, which is typically used to evaluate spatial neglect but also provides information about hemispheric functioning [92]. However, in the two included studies that utilized this specific task, bipolar participants exhibited differences in terms of performance [67,77].

Reaction time and processing speed have also been widely examined in neuropsychological studies of BD. Atagun et al. found that stronger lateralization correlated with faster processing in bipolar patients, possibly compensating for delays in interhemispheric transmission [42]. Similarly reduced interhemispheric summation was reported, while using fMRI, Caligiuri et al. showed that manic and depressed bipolar patients failed to properly inhibit activity in the supplementary motor area, resulting in poorer reaction time performance compared with controls [47,53].

Several studies employing neuroimaging methods have explored visuospatial and visual processing in BD. Bruder et al. found that bipolar patients performed worse in visuospatial tasks presented in the left visual field, suggesting right hemisphere dysfunction [45]. Similarly, Egorov and Nikolaenko observed mood-related differences in hemispheric activation: depressed patients showed stronger right hemisphere activation, while manic patients exhibited increased left hemisphere activation and poorer spatial accuracy, indicating reduced right hemisphere involvement during mania [51]. Overall, these findings support the important role of the right hemisphere in perceptual integration.

Regarding motor tasks, Lyon et al. reported that both right- and left-handed bipolar patients tended to turn left in the Lyon two-choice task and pin test, unlike healthy right-handed individuals who turned right [63]. This pattern suggests a disruption in hemispheric dominance controlling movement direction and hand preference, possibly linked to increased left dopaminergic activity in the striatum. Bipolar patients also showed slower performance, which may reflect prefrontal or basal ganglia dysfunction or medication effects. 

A smaller number of studies examined language network function and asymmetry in BD using neuropsychological measures. Coffman et al. found nonverbal memory deficits in bipolar patients but no differences in verbal memory or structural/functional asymmetry, suggesting that BD cannot be characterized solely as a right hemisphere disorder [48]. Padovan et al. reported reduced left hemisphere lateralization during language and phonemic tasks, accompanied by decreased functional connectivity in dorsal attention networks, although overall language performance remained comparable to controls [72].

Several studies have examined functional motor and sensory asymmetry in BD. Lohr and Caligiuri found motor asymmetries in bipolar patients, as greater left-hand dysfunction and right hemisphere involvement was exhibited [47,62]. Likewise, studies support differentiated motor and cognitive speed processing accompanied by stronger lateralization and potential deficits, likely reflecting delays in interhemispheric transmission [42,63,83].

In conclusion, the available evidence highlights substantial heterogeneity in structural and functional hemispheric asymmetry in BD. Findings are both convergent and contradictory, underscoring the complexity of hemispheric organization in the disorder and the need for more integrative and methodologically consistent research. 

## 4. Discussion

**Figure 2:**
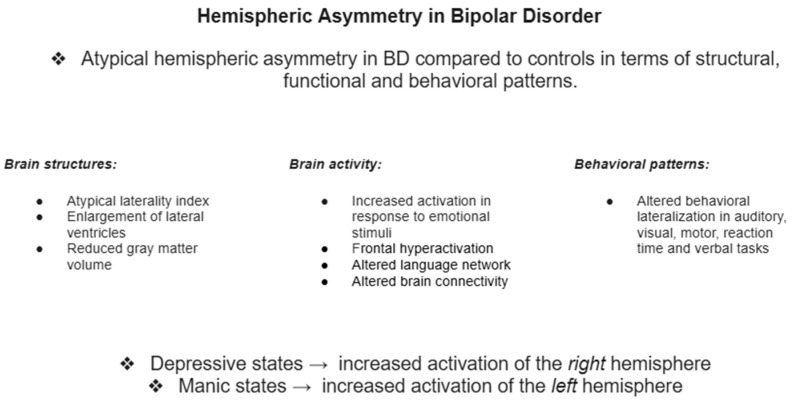
Schematic summary of key conclusions.

This systematic review aimed to identify potential patterns of structural and functional brain asymmetry in individuals with BD compared to healthy controls. It also seeks to explore potential trends in how the left and right hemispheres function in BD and how these patterns relate to pathological mood shifts. By categorizing findings into structural, functional-neuroimaging and neuropsychological domains, key conclusions are highlighted for each category. The review further connects these observations to the broader theoretical framework of BD and brain asymmetry, discusses potential limitations and directions for future research aimed at addressing gaps in the literature, and improves understanding of the topic in question. A schematic summary of the findings can be found in Figure 2.

### 4.1. Structural Neuroimaging Findings

A detailed review of structural neuroimaging findings on brain asymmetry in BD reveals substantial heterogeneity in the literature. Overall, no consistent evidence identifies specific brain regions that show reliable morphological asymmetry in bipolar patients compared to healthy controls, making generalization difficult.

However, one finding that appears relatively consistent is ventricular enlargement in BD [30,64,65,73]. Some studies suggest greater enlargement in the left hemisphere, and the literature reports associations between ventricular enlargement, depressive symptoms, disease duration, and the number of bipolar episodes [74,93,94,95,96]. This observation aligns with the ‘Valence Hypothesis’, which posits that the left hemisphere primarily processes positive emotions [8]. Consequently, left hemisphere atrophy or ventricular enlargement may contribute to depressive symptoms [6]. Nevertheless, findings remain inconsistent, as Kieseppä et al. did not observe ventricular enlargement in bipolar patients [58].

Another relatively consistent finding across studies is a general reduction in gray matter volume in BD, observed in regions such as the cingulate cortex, superior temporal gyrus, parietal lobe, and temporal lobe [28,52,64,75]. The cingulate gyrus plays a central role in reward processing, conflict monitoring, and empathy; therefore, reduced gray matter in this area may disrupt emotional processing. The temporal lobes, particularly the superior temporal gyrus, are crucial for interpreting emotional prosody and social cues. Right temporal damage can impair emotional perception and nonverbal communication, whereas left temporal damage may affect emotional expression. The parietal lobe also contributes to emotional decision-making, organization, and regulation [97]. Reductions in gray matter within these emotion-related regions may therefore contribute to mood dysregulation in BD. However, conflicting findings exist; for instance, Pinto et al. reported increased gray matter in certain right hemisphere regions, including the premotor cortex, temporo-occipital cortex, ventral prefrontal cortex, and anterior cingulate cortex [75]. These alterations may be associated with hyperactivation of the right hemisphere and mood disturbances [25]. Overall, structural changes in gray matter within emotion-related brain regions appear to play a significant role in the symptomatology of BD.

Findings from diffusion tensor imaging (DTI) studies, particularly those examining fractional anisotropy (FA), indicate that individuals with BD show reduced FA in several brain regions and connections, including the corpus callosum, interhemispheric fibers in anterior and subcortical areas, and right hemisphere occipito-parietal pathways [55,91]. Reduced FA in the right hemisphere is frequently reported in the literature [98,99,100], alongside a general decrease in FA across various brain regions in BD [98,101,102,103,104]. Conversely, increased radial and mean diffusivity in the right hemisphere have also been observed [55], a finding supported by several studies [102,105,106,107,108,109,110]. These patterns suggest disrupted connectivity in the right hemisphere, which may underlie difficulties in processing emotional information. Given that the right hemisphere primarily mediates contextual and nonverbal communication, impaired intrahemispheric communication could lead to poorer integration of emotional experiences, potentially contributing to both manic and depressive episodes in BD [111,112].

White matter volume findings in BD have been highly variable, making it difficult to identify consistent patterns of increase, decrease, or stability relative to healthy controls [28,52,58,60,64,70]. While DTI studies indicate altered white matter connectivity, volumetric studies have not produced equally consistent results. Emerging evidence suggests that dysfunction of the glymphatic system may indicate a novel pathophysiological mechanism contributing to white matter alterations in psychiatric disorders, including BD. As observed in neuroimaging studies, impaired clearance of metabolic waste has been connected with myelin alterations, neuroinflammatory processes, and white matter disruption, potentially offering a framework of a unifying biological mechanism underlying the inconsistent structural and connectivity findings reported across studies [113]. Research on subcortical structures such as the amygdala, thalamus, hypothalamus, and hippocampus remains limited and inconclusive regarding hemispheric asymmetry in BD. Nevertheless, these structures likely play important roles in emotional processing. The hippocampus links emotionally salient experiences to their contextual representations, and its altered function may contribute to memory and cognitive deficits in BD [24,114,115]. Altered hippocampal structure is also connected to changes in neuroplasticity, as essential neuroplasticity markers like BDNF have been found to be reduced in BD patients [27]. The thalamus mediates emotional and peripheral sensory information, and functional alterations may affect mood regulation [116]. Although the thalamus is not exclusively involved in emotional processing, its disruption could influence emotional responses. The amygdala evaluates emotional valence, with its central nucleus serving as a key relay to hypothalamic centers [117,118,119]. Schindler et al. reported increased hypothalamic volume in the left hemisphere among individuals with bipolar or other mood disorders, suggesting that increased left hypothalamic activity could lead to overactivation of the left amygdala and potentially contribute to manic symptoms in BD [81].

Studies examining the integrity of the cerebral cortex and lobes in BD show considerable heterogeneity, with inconsistent findings regarding the temporal, occipital, and parietal lobes [64,73,76]. However, there is relatively consistent evidence of structural abnormalities in the frontal lobe and anterior brain regions, including bilateral volume reductions and other anomalies [39,48,91]. The frontal cortex plays a crucial role in executive control, behavioral regulation, and higher-order cognitive processes. Structural changes and connectivity disruptions within the frontal lobes have been linked to deficits in emotion regulation, cognitive control, and behavioral inhibition in BD [29,120,121,122,123,124,125,126]. Progressive frontal abnormalities have also been associated with manic symptoms, likely due to the frontal lobe’s involvement in the brain’s reward system, where damage or functional disruptions may lead to impulsivity, increased goal-directed behavior, and euphoric mood characteristic of mania [39,127]. Thus, regardless of hemispheric asymmetry, frontal lobe alterations appear to play a key role in bipolar symptomatology through their widespread brain connections.

A key conclusion emerging from the literature, regardless of the specific structural differences reported, is the consistent presence of atypical brain asymmetry in BD [43,48,52,55,60,65,75,89]. Despite the heterogeneity and occasional contradictions across studies, most converge on the observation of atypical hemispheric asymmetry, often characterized by increased rightward asymmetry in individuals with BD [55,75,89]. This atypical hemispheric organization may disrupt normal interhemispheric communication, thereby contributing to dysregulation of cognitive and emotional functions [128,129,130]. Right hemisphere dominance, which has also been reported in conditions such as autism spectrum disorder [131,132,133] and schizophrenia [134,135], may lead to increased processing of negative emotional stimuli and nonverbal communication cues. At the same time, relative underactivity of the left hemisphere may impair reward processing and goal-directed planning—features commonly observed in bipolar symptomatology [112,136,137].

Mood states in BD appear to be associated with patterns of brain asymmetry, although not all studies examined differences between depressive and manic phases. Evidence suggests that structural brain variations, including increased left hemisphere perfusion [40], reduced cortical volume [28], frontal lobe reductions [39], ventricular enlargement [65], and increased hemispheric asymmetry [60], may relate to mania, depression, or mood fluctuations. These findings support the notion of state-dependent hemispheric lateralization, consistent with classical emotion lateralization theories. Specifically, the Right Hemisphere [2] and ‘Valence Hypothesis’ [8] theories, Davidson’s Approach–Withdrawal model [138], and recent reviews [35] suggest that left hemisphere dominance is associated with approach behavior and manic states, while right hemisphere dominance is more closely linked to withdrawal behavior and depressive symptoms in BD.

### 4.2. Functional Neuroimaging Findings

As mentioned in the Section 3, part of the findings concerns functional neuroimaging studies. Within this broader framework, considerable heterogeneity was observed, limiting the extent to which the results can be generalized. More specifically, findings regarding the frontal cortex demonstrate substantial variability. Studies report differences in both the functions examined and the localizations of frontal and prefrontal brain regions, making any generalization difficult. Nevertheless, some evidence suggests increased activation of left frontal brain regions during manic states [44,71].

With respect to the amygdala, findings also demonstrate substantial heterogeneity. Although hyperactivation of the right amygdala has been reported, the available evidence is insufficient to support a definitive conclusion [66]. In relation to emotional processing, a correlation between positive emotion and activation of the left hemisphere has been reported [82]. Within the theoretical framework outlined in the Introduction, this finding appears to contradict the ‘Valence Hypothesis’ [8]. However, the available evidence remains insufficient for a generalization of this interpretation.

Furthermore, although several studies indicate the presence of an atypical language network, the evidence does not support a unified conclusion, as studies employ different tasks and investigate different brain regions. Some studies report reduced or absent lateralization, whereas others report no differences compared to non-bipolar individuals [72,79,80,86]. Nevertheless, there appears to be some convergence toward the notion of atypical functioning within the language network in BD.

A similar pattern is observed in studies examining the auditory cortex. Although two studies reported no asymmetry in the topographical location of M100 and M200 components, other findings concern diverse functions and regions, making interpretation difficult [87,88]. Comparable variability is also evident in studies examining cerebral asymmetry in the temporal cortex, motor cortex and attention networks.

### 4.3. Functional Findings from Neuropsychological Assessment

Neuropsychological studies investigating functional hemispheric asymmetries in BD demonstrate greater consistency than morphofunctional imaging findings, suggesting a relatively stable pattern of cognitive dysfunction in these patients.

Some consistent trends emerge from dichotic listening paradigms. These studies mainly suggest an abnormal left-ear shift during manic and euthymic states, indicating right hemisphere hyperactivation. Following recovery, a shift toward the typical left-ear advantage (LEA), typical of normal asymmetry, is observed [45,46,57,68]. According to Najt et al., atypical LEA in emotional prosody tasks may represent a neurobehavioral vulnerability marker of emotional dysregulation, reflecting a functional breakdown in the right fronto-temporal network in both symptomatic and euthymic BD [68]. LEA implying atypical right hemisphere hyperactivation was highlighted primarily in BD patients during manic states, and to a lesser extent in euthymic states. Consequently, reduced hemispheric laterality, which appears to vary depending on mood state, may significantly affect performance in auditory laterality tasks. Indeed, BD patients in euthymic, manic, or depressive states exhibit atypical laterality responses to auditory stimuli [51,78]. Additional studies suggest that mood states may functionally ”bias” hemispheric activity [17,139,140,141]. Other researchers have emphasized the prominent role of the right hemisphere in both emotional and cognitive processing [142,143]. Accordingly, affective state-dependent hyperactivation of the non-dominant hemisphere, combined with functional regression of the dominant hemisphere, has been proposed as a possible mechanism underlying manic episodes, potentially accompanied by impairments in the comprehension of emotional language cues [144].

Less consistent findings regarding spatial attention in BD come from studies using the line bisection paradigm. Although some studies report atypical line bisection performance in BD patients, no systematic pattern of bias has been identified [67,77]. In this regard, part of the studies examining visuospatial perception in BD patients similarly show heterogeneous findings, preventing firm conclusions [69]. Nevertheless, performance in these tasks may suggest right hemisphere hyperactivation during depressive states and increased left hemisphere activation during manic states [44,45,50,71].

In addition, studies examining speed and reaction time tasks report relatively slower responses in BD patients compared to healthy controls [32,42,47,53]. Most authors interpreted this delay as reflecting dysfunction in functional lateralization and interhemispheric communication.

Moreover, several neuropsychological studies have examined hemispheric asymmetry in relation to emotional tone and responses to positively valenced stimuli [145]. However, these findings remain highly variable across both morphofunctional and neuropsychological approaches and therefore do not allow firm conclusions. A similar pattern is observed in studies examining language network lateralization in BD, and those investigating motor function and the motor cortex [62]. In these domains, findings either support or contradict existing theories of hemispheric specialization for emotional processing.

### 4.4. Limitations

The present systematic review is subject to several limitations that also characterize the broader literature on cerebral asymmetry in BD. The primary obstacle to integrating morphofunctional and neuropsychological findings is the high degree of heterogeneity among studies. A major issue during the process of identifying eligible studies was also the fact that only a limited number of them were integrating both components of the present review. Another barrier to generalization is the relatively small sample size in the majority of studies. At the same time, various studies included participants with different psychiatric disorders, most commonly schizophrenia and major depressive disorder. In some cases, it was unclear whether the reported findings were specific to BD or shared with other psychopathological conditions. In other instances, comparison groups (e.g., schizophrenia or depression) were substantially larger than the BD samples. Furthermore, many studies differed in their diagnostic approaches to BD. In some cases, samples were stratified according to BD subtype (I vs. II), while in others this distinction was not made. Similarly, although mood states (manic, depressive, euthymic) are highly relevant to the disorder, studies varied considerably in whether and how these states were considered. Finally, individual differences, which may influence the course and expression of the disorder, were rarely taken into account. Taken together, the aforementioned limitations currently prevent a comprehensive integration of findings regarding cerebral asymmetry in BD.

## 5. Conclusions

In summary, the existing literature on structural and functional cerebral asymmetry in BD is characterized by substantial heterogeneity. The initial hypotheses are only partially supported, as the majority of the studies report evidence suggesting atypical cerebral asymmetry in BD patients. Likewise, a significant proportion of studies also supports the notion of relative hyperactivation of the left hemisphere during manic states and of the right hemisphere during depressive states. However, consistent agreement across studies has not yet been achieved, and the generalization of findings remains limited due to contradictory evidence. As a result, future research on cerebral asymmetry in BD is essential in order to address the significant gaps in the current literature and to clarify the neurobiological mechanisms underlying the disorder. In this context, it is suggested that future studies should incorporate more systematic methodological stratification, particularly with respect to mood state, bipolar disorder subtype, duration of illness, as well as medication status, as these factors may critically influence observed asymmetry patterns. Taking such group differences into consideration might reduce heterogeneity and enhance the comparability of findings. Future investigations may also benefit from stronger theoretical guidance, particularly from established models of emotional hemispheric asymmetry, in order to better integrate neurobiological data with theoretical frameworks.

## Figures and Tables

**Table 1:** Table of results from the 56 studies included.

Study	Sample	Method	Direction of Asymmetry/Location of Impairments	Type of Asymmetry
Abé [39]	BD (*n* = 31) [at least one manic episode (*n* = 13), non-mania (*n* = 18)]	MRI	• BD M: frontal irregularities	Structural asymmetry
Agarwal [40]	BD (*n* = 14): BD Ι (*n* = 10), BD ΙΙ (*n* = 4)	MRI	• BD: atypical cerebral blood lateralization	Structural asymmetry
Altshuler [41]	BD (*n* = 11), CG (*n* = 17)	fMRI, neuropsychological assessment	• BD: left ventral lateral prefrontal cortex activation • BD: right dorsolateral prefrontal cortex activation	Functional asymmetry
Atagun [42]	BD Ι EU (*n* = 68), CG (*n* = 65)	Motor and cognitive processing speed assessment (including memory, visuospatial functions, and hemispheric lateralization)	• Hemispheric lateralization, cognitive processing and speed	Functional asymmetry (behavioral lateralization)
Bellani [32])	BD (*n* = 16), CG (*n* = 40)	Neuropsychological assessment, visual stimuli reaction time	• BD: no interhemispheric information transfer dysfunction • BD: no slowed reaction to visual stimuli with right hand	Functional asymmetry (visual asymmetry and interhemispheric asymmetry)
Bilder [43]	BD (*n* = 20), CG (*n* = 67), other disorders (*n* = 148)	MRI	• Laterality index: BD > CG and other disorders	Structural asymmetry
Blumberg [44]	BD Ι, Μ and ΕU, (*n* = 11)	PET	• Mania: increased activity of LH dorsal anterior cingulate cortex (dACC) and the caudate nucleus, activity in frontal lobe neural networks	Functional asymmetry (hyperactivation)
Bruder [45]	BD (*n* = 11), CG (*n* = 24)	ERP, visual tests and dichotic listening test	• L visual field, not R • BD reduced N100 amplitude for stimuli in L visual field	Functional asymmetry (electrophysiological, visual και auditory asymmetry)
Bruder [46]	BD Μ and ΕU (*n* = 35), CG (*n* = 26)	Dichotic listening test	• Impairment in processing complex tones in L ear	Functional asymmetry (auditory asymmetry)
Caligiuri [47]	BD (*n* = 18) [DP (*n* = 6), Μ (*n* = 12)], CG (*n* = 13)	fMRI, reaction speed test	• Primary motor cortex (Μ1) asymmetry • Increased activation in L Μ1	Functional asymmetry (sensorimotor asymmetry)F
Coffman [48]	BD with psychotic symptoms (*n* = 30), CG (*n* = 52)	MRI (in 29 BD and 25 CG), psychometric tests	• No functional or structural asymmetry found • BD: superficially extended RH damage	Functional and structural asymmetry
Dewan [49]	BD (*n* = 25), CG (*n* = 25)	CT scans, neuropsychological assessment	• No differences in asymmetry found	Functional and structural asymmetry
Egoroν [50]	BD (*n* = 18) [Μ (*n* = 18)–DP syndromes (*n* = 29)], CG (*n* = 30)	Visuospatial drawing test	• BD DP: upper L field preference • BD Μ: upper R field preference	Functional asymmetry (visuospatial asymmetry and completion perception)
Egorov [51]	BD (*n* = 56) [DP (*n* = 32), Μ (*n* = 24)]	Auditory and visual test (a: measurement of poststimulatory auditory adaptation (PAA) on the left and right ears; b: measurement of after-image thresholds (AITs) in the left and right visual fields)	• BD DP: PAA R ear > L AIT R visual field < L • BD Μ: PAA L ear > R AIT L visual field < R	Functional asymmetry (auditory and visual asymmetry)
Ferro [52]	BD (*n* = 38), CG (*n* = 42)-Longitudinally: BD(*n* = 17), CG(*n* = 16)	MRI	• Parietal lobe white and gray matter: BD < CG • BD: parietal lobe white matter RH = LH • CG: parietal lobe white matter RH< LH	Structural asymmetry
Florio [53]	BD I (*n* = 49), CG (*n* = 44)	Visual stimuli test and reaction time	• Right hand reaction time: BD = CG	Functional asymmetry (interhemispheric integration)
Harmon-Jones [54]	BD (*n* = 41), CG (*n* = 53)	EEG	• BD: increased frontal activation in demanding goal-orientated tests in LH ≠ RH	Functional asymmetry (task demand)
Ho [55]	BD (*n* = 35), CG (*n* = 77), SHZ (*n* = 150)	DTI	• Increase in RH radial diffusivity (RA) in BD • Increase in mean diffusivity (MD) of RH areas in BD • Increase in leftward laterality index (LI) in BD	Structural asymmetry
Javadapour [56]	BD (*n* = 24), CG (*n* = 24)	MRI	• Left hippocampus: BD > CG (8.5%)	Structural asymmetry
Kaprinis [57]	BD (*n* = 26), CG (*n* = 30)	Dichotic listening test	• L ear: BD Μ > CG • BD ΕU no laterality deficiencies	Functional asymmetry (auditory asymmetry)
Kieseppä [58]	Twins BD Ι (*n* = 24), healthy twins of BD (*n* = 15), CG (*n* = 27)	MRI, interview	• LH white matter bd and twins < CG • RH white matter < twins and CG	Structural asymmetry (white matter)
Koller-Schlaud [59]	BD (*n* = 22), CG (*n* = 32)	EEG, emotional face stimuli	• Alpha-1 asymmetry scores did not differ between BD with or without DP	Functional asymmetry (emotional processing)
Li [60]	BD (*n* = 49), CG (*n* = 55)	DTI	• BD: atypical asymmetry in feeder connections and local connections	Structural asymmetry
Li [61]	BD (*n* = 48), CG (*n* = 48)	MRI	• BD: increased laterality index in attention network	Structural asymmetry
Lohr [62]	BD (*n* = 13), CG (*n* = 50), SHZ (*n* = 23)	Motor skills test	• BD: L hand dysfunction	Functional asymmetry (motor coordination)
Lyon [63]	BD (*n* = 19), CG (*n* = 19)	Neuropsychological Tests (Lyon two-choice task, visuomotor sequence test-pin test)	• BD: tendency for leftward rotation ≠ CG: τendency for rightward rotation	Functional asymmetry (visual and motor asymmetry)
Maller [64]	BD (*n* = 35) [Ι (*n* = 17), ΙΙ (*n* = 18)], CG (*n* = 36)	MRI	• Gray matter, hippocampus, occipital lobe volume: BD < CG • Lateral ventricles volume: BD > CG • Occipital bending towards RH: BD > CG	Structural asymmetry
Manelis [65]	BD (*n* = 38) [BD Ι (*n* = 11), BD ΙΙ (*n* = 27)], DP (*n* = 57), CG (*n* = 54)	MRI	• Lateral ventricles volume: BD and DP > CG	Structural asymmetry
Moorhead [28]	BD Ι (*n* = 21), CG (*n* = 20)	MRI	• LH hippocampus gray matter, cerebellum and fusiform gyrus decrease: BD > CG • No white matter differences BD and CG	Structural asymmetry
Najt [66]	BD Ι EU (*n* = 13), CG (*n* = 15)	fMRI	• BD: Differences in connectivity of sensory organs towards R amygdala • No differences from or towards L amygdala • Activation in posterior cingulate gyrus, superior temporal gyrus, calcarine fissure → R amygdala decreased activation	Functional asymmetry
Najt [67]	BD Ι (*n* = 22), CG (*n* = 18)	Line-bisection task	• BD ΕU: decreased leftward bisection direction with L hand • Direction of bisection: BD > CG (closer to line center)	Functional asymmetry (hand preference and visuomotor skills)
Najt [68]	BD Ι EU(*n* = 22), CG (*n* = 18)	Dichotic listening test	• Emotional prosody: BD ΕU → atypical hemispheric functional asymmetries • BD ΕU did not present left ear advantage • Verbal test: Right ear advantage → BD = CG	Functional asymmetry (emotional prosody)
Nikolaenko [69]	First case: DP (BD and non-BD) (*n* = 62), SHZ (*n* = 30) Second case: patients (*n* = 90) Third case: DP (BD and non BD) (*n* = 35), Μ (*n* = 22)	Line drawing analysis (Rausehenbach)	• Study of drawings in DP → RH dominance • Study of drawings in Μ → LH dominance	Functional asymmetry (visuospatial asymmetry)
Nugent [70]	BD (*n* = 36) [in treatment (*n* = 20), no treatment (*n* = 16)], CG (*n* = 65)	MRI	• BD in or not in treatment: decreased gray matter (differences in regions) • BD: increased white matter in posterior cingulate cortex	Structural asymmetry
Nusslock [71]	BD (*n* = 58), CG (*n* = 59)	EEG, interview, behavioral assessment	• EEG: LH central frontal activation → BD HΜ > CG and BD DP	Functional asymmetry
Padovan [72]	BD (*n* = 18), CG (*n* = 16)	fMRI, neuropsychological tests	• Verbal fluency: BD = CG • Mental rotation: BD < CG • Verbal tests: impaired L lateralization in BD	Functional asymmetry (verbal lateralization)
Pearlson [73]	BD (*n* = 27), CG (*n* = 60), SHZ (*n* = 46)	MRI	• L anterior superior temporal gyrus: BD > CG • LH amygdala: BD < CG	Structural asymmetry
Pettigrew [74]	BD EU (*n* = 18), CG (*n* = 49)	Experimental tests, binocular rivalry test	• BD: slow rate of interhemispheric information switching	Functional asymmetry (interhemispheric switching)
Pinto [75]	BD (*n* = 20), CG (*n* = 20), SHZ (*n* = 20)	MRI	• RH gray matter volume: BD > CG• RH asymmetry: BD → middle frontal gyrus, anterior cingulate cortex	Structural asymmetry
Radonić [76]	BD (*n* = 15), CG (*n* = 15), SHZ (*n* = 15), schizoaffective disorder (*n* = 15)	MRI	• BD: LH temporal lobe < RH	Structural asymmetry
Rao [77]	BD (*n* = 31), CG (*n* = 103), SHZ (*n* = 30)	Line-bisection task	• Line bisection: BD and SHZ < CG • BD: leftward deviation with R hand	Functional asymmetry (hand preference and visuomotor skills)
Reite [78]	BD ΕU (*n* = 17), CG (*n* = 17)	MEG, auditory stimuli	• BD: atypical lateralization of stage gamma band in primary auditory cortex • BD: typical asymmetry in secondary auditory cortex (R frontal → L)	Functional asymmetry (auditory asymmetry)
Romeo [79]	BD ΕU (*n* = 18), CG (*n* = 16)	fMRI	• BD: atypical LH spatial map of the language network, atypical increased RH activation	Functional asymmetry (language network)
Royer [80]	BD (*n* = 20), CG BD (*n* = 32), SHZ (*n* = 31), CG SHZ (*n* = 31)	fMRI, verbal task	• BD: gray matter volume asymmetry in language network → not decreased	Functional asymmetry (language network)
Schindler [81]	BD (*n* = 21), CG (*n* = 23), DP without treatment (*n* = 20), DP in treatment (*n* = 20)	MRI	• Increased hypothalamus volume in BD and DP: LH > RH	Structural asymmetry
Spironelli [82]	BD ΕU (*n* = 17), DP (*n* = 25), dysthymia (*n* = 21)	EEG	• BD ΕU: frontal asymmetry, increased LH activation	Functional asymmetry
Strakowski [30]	BD (*n* = 24), CG (*n* = 22)	MRI	• BD: LH lateral ventricles enlargement	Structural asymmetry
Stratta [83]	BD (*n* = 13), CG (*n* = 15)	Quality extinction test	• BD: difficulty in tactile recognition with R hand ≠ L hand	Functional asymmetry (tactile recognition)
Sukno [84]	BD (*n* = 22), SHZ (*n* = 22), CG (*n* = 49)	3D laser imaging	• Face asymmetry: BD ≠ CG	Structural asymmetry
Tas [85]	BD (*n* = 25), DP (*n* = 56)	EEG	• BD: atypical and altered patterns of RH functional connectivity and interhemispheric connectivity	Functional asymmetry
Tréhout [86]	BD (*n* = 20), SHZ (*n* = 20), CG (*n* = 40)	fMRI: functional lateralization index (FLI)	• BD: decreased left lateralization in speech area	Functional asymmetry
Wang [87]	BD (*n* = 20), SHZ (*n* = 20), CG (*n* = 20)	MEG	•Symmetry in source location of the M100 response in BD ≠ CG	Functional asymmetry
Wang [88]	BD (*n* = 26), SHZ (*n* = 24), CG (*n* = 31)	MEG	•Symmetric M100 and Μ200 auditory response pattern in RH superior temporal in BD ≠ CG	Functional asymmetry (auditory asymmetry)
Wang [89]	BD (*n* = 49), CG (*n* = 61)	MRI	• BD: reduced R frontal, parietal, occipital and temporal lobe asymmetry	Structural asymmetry
Wei [90]	BD (*n* = 29) [first episode DP (*n* = 16), first episode Μ (*n* = 13)], CG (*n* = 30)	fMRI	• Functional connectivity in resting state between amygdala and L orbitofrontal cortex: BD < CG	Functional asymmetry
Yamada [91]	BD (*n* = 20), DP (*n* = 18), CG (*n* = 21)	MRI, DTI	• BD and DP: microstructural irregularities in corpus callosum anterior fibers → bilateral connection with frontal lobe	Structural asymmetry

Note: CG—Control Group; BD—Bipolar Disorder (I or II); EU—Euthymia; SHZ—Schizophrenia; LH—Left Hemisphere; RH—Right Hemisphere; M—Mania; DP—Depression.

## Data Availability

The original contributions presented in this study are included in the article/Supplementary Materials. Further inquiries can be directed to the corresponding author.

## Supplementary Materials

The following supporting information can be downloaded at: https://www.mdpi.com/article/10.3390/medicina62040792/s1, Table S1: Detailed risk of bias assessment of included studies, PRISMA 2020 Main Checklist.

## References

[1] Broca P. (1861). Remarks on the Seat of the Faculty of Articulated Language, Following an Observation of Aphemia (Loss of Speech). Bull. Soc. Anat..

[2] Gainotti G. (2020). Emotions and the Right Side of the Brain.

[3] Hughlings J. (1898). The Hughlings Jackson Lecture on the Relations of Different Divisions of the Central Nervous System to One Another and to Parts of the Body. Lancet.

[4] Gainotti G. (2000). Neuropsychological Theories of Emotion. The Neuropsychology of Emotion.

[5] Borod J.C., Cicero B.A., Obler L.K., Welkowitz J., Erhan H.M., Santschi C., Grunwald I.S., Agosti R.M., Whalen J.R. (1998). Right Hemisphere Emotional Perception: Evidence Across Multiple Channels. Neuropsychology.

[6] Gainotti G. (2019). Emotions and the Right Hemisphere: Can New Data Clarify Old Models?. Neuroscientist.

[7] Mandal M.K., Ambady N. (2004). Laterality of Facial Expressions of Emotion: Universal and Culture-Specific Influences. Behav. Neurol..

[8] Reuter-Lorenz P., Davidson R.J. (1981). Differential Contributions of the Two Cerebral Hemispheres to the Perception of Happy and Sad Faces. Neuropsychologia.

[9] Stuss D.T., Gow C.A., Hetherington C.R. (1992). “No Longer Gage”: Frontal Lobe Dysfunction and Emotional Changes. J. Consult. Clin. Psychol..

[10] Davidson R.J., Ekman P., Saron C.D., Senulis J.A., Friesen W.V. (1990). Approach-Withdrawal and Cerebral Asymmetry: Emotional Expression and Brain Physiology: I. J. Pers. Soc. Psychol..

[11] Gainotti G. (2019). The Role of the Right Hemisphere in Emotional and Behavioral Disorders of Patients with Frontotemporal Lobar Degeneration: An Updated Review. Front. Aging Neurosci..

[12] Barr L.K., Kahn J.H., Schneider W.J. (2008). Individual Differences in Emotion Expression: Hierarchical Structure and Relations with Psychological Distress. J. Soc. Clin. Psychol..

[13] Wyble B., Sharma D., Bowman H. (2008). Strategic Regulation of Cognitive Control by Emotional Salience: A Neural Network Model. Cogn. Emot..

[14] Champagne-Lavau M., Joanette Y. (2009). Pragmatics, Theory of Mind and Executive Functions After a Right-Hemisphere Lesion: Different Patterns of Deficits. J. Neurolinguist..

[15] Tsolakopoulos D., Kasselimis D., Laskaris N., Angelopoulou G., Papageorgiou G., Velonakis G., Varkanitsa M., Tountopoulou A., Vassilopoulou S., Goutsos D. (2023). Exploring Pragmatic Deficits in Relation to Theory of Mind and Executive Functions: Evidence from Individuals with Right Hemisphere Stroke. Brain Sci..

[16] Weed E. (2008). Theory of Mind Impairment in Right Hemisphere Damage: A Review of the Evidence. Int. J. Speech-Lang. Pathol..

[17] Gainotti G. (1972). Emotional Behavior and Hemispheric Side of the Lesion. Cortex.

[18] Alemà D. (1960). Sulle Modificazioni Cliniche Ed Elettroencefalografiche da Introduzione Intracarotidea di Iso-Amil-Etil-Barbiturato di Sodio Nell’uomo. Boll. Soc. Ital. Biol. Sper..

[19] Perria L., Rosadini G., Rossi G.F. (1961). Determination of Side of Cerebral Dominance with Amobarbital. Arch. Neurol..

[20] Terzian H. (1958). Cecotto Su Un Nuovo Metodo per la Determinazione e lo Studio Della Dominanza Emisferica. G. Psichiatr. Neuropatol..

[21] American Psychiatric Association (2013). Diagnostic and Statistical Manual of Mental Disorders.

[22] Kring A.M., Johnson S.L., Davison G.C., Neale J.M. (2014). Abnormal Psychology.

[23] De Bartolomeis A., Buonaguro E.F., Iasevoli F., Tomasetti C. (2014). The Emerging Role of Dopamine–Glutamate Interaction and of the Postsynaptic Density in Bipolar Disorder Pathophysiology: Implications for Treatment. J. Psychopharmacol..

[24] Frey B.N., Andreazza A.C., Nery F.G., Martins M.R., Quevedo J., Soares J.C., Kapczinski F. (2007). The Role of Hippocampus in the Pathophysiology of Bipolar Disorder. Behav. Pharmacol..

[25] Lim C.S., Baldessarini R.J., Vieta E., Yucel M., Bora E., Sim K. (2013). Longitudinal Neuroimaging and Neuropsychological Changes in Bipolar Disorder Patients: Review of the Evidence. Neurosci. Biobehav. Rev..

[26] Strakowski S.M., DelBello M.P., Adler C.M. (2005). The Functional Neuroanatomy of Bipolar Disorder: A Review of Neuroimaging Findings. Mol. Psychiatry.

[27] Monteleone P., Serritella C., Martiadis V., Maj M. (2008). Decreased Levels of Serum Brain-Derived Neurotrophic Factor in both Depressed and Euthymic Patients with Unipolar Depression and in Euthymic Patients with Bipolar I and II Disorders. Bipolar Disord..

[28] Moorhead T.W.J., McKirdy J., Sussmann J.E.D., Hall J., Lawrie S.M., Johnstone E.C., McIntosh A.M. (2007). Progressive Gray Matter Loss in Patients with Bipolar Disorder. Biol. Psychiatry.

[29] Savitz J.B., Price J.L., Drevets W.C. (2014). Neuropathological and Neuromorphometric Abnormalities in Bipolar Disorder: View from the Medial Prefrontal Cortical Network. Neurosci. Biobehav. Rev..

[30] Strakowski S.M., DelBello M.P., Sax K.W., Zimmerman M.E., Shear P.K., Hawkins J.M., Larson E.R. (1999). Brain Magnetic Resonance Imaging of Structural Abnormalities in Bipolar Disorder. Arch. Gen. Psychiatry.

[31] Bearden C.E., Hoffman K.M., Cannon T.D. (2001). The Neuropsychology and Neuroanatomy of Bipolar Affective Disorder: A Critical Review. Bipolar Disord..

[32] Bellani M., Marzi C.A., Savazzi S., Perlini C., Cerruti S., Ferro A., Marinelli V., Sponda S., Rambaldelli G., Tansella M. (2010). Laterality Effects in Schizophrenia and Bipolar Disorder. Exp. Brain Res..

[33] Mundorf A., Borawski J., Ocklenburg S. (2023). Behavioral Lateralization in Bipolar Disorders: A Systematic Review. Int. J. Bipolar Disord..

[34] Quraishi S., Frangou S. (2002). Neuropsychology of Bipolar Disorder: A Review. J. Affect. Disord..

[35] Moebus L., Quirin M., Ehrlenspiel F. (2023). Cerebral Asymmetry in Bipolar Disorders: A Scoping Review. Biol. Psychol..

[36] Higgins J.P.T., Green S. (2008). Assessing Risk of Bias in Non-Randomized Studies. Cochrane Handbook for Systematic Reviews of Interventions.

[37] Wells G., Shea B., O’Connell D., Peterson J., Welch V., Losos M., Tugwell P. (2014). Newcastle-Ottawa Quality Assessment Scale Cohort Studies. University of Ottawa. https://www.ncbi.nlm.nih.gov/books/NBK56664/bin/appc-fm3.pdf.

[38] Page M.J., McKenzie J.E., Bossuyt P.M., Boutron I., Hoffmann T.C., Mulrow C.D., Shamseer L., Tetzlaff J.M., Akl E.A., Brennan S.E. (2021). The PRISMA 2020 Statement: An Updated Guideline for Reporting Systematic Reviews. BMJ.

[39] Abé C., Ekman C.-J., Sellgren C., Petrovic P., Ingvar M., Landén M. (2015). Manic Episodes Are Related to Changes in Frontal Cortex: A Longitudinal Neuroimaging Study of Bipolar Disorder 1. Brain.

[40] Agarwal N., Bellani M., Perlini C., Rambaldelli G., Atzori M., Cerini R., Vecchiato F., Pozzi Mucelli R., Andreone N., Balestrieri M. (2008). Increased Fronto-Temporal Perfusion in Bipolar Disorder. J. Affect. Disord..

[41] Altshuler L., Bookheimer S., Townsend J., Proenza M.A., Sabb F., Mintz J., Cohen M.S. (2008). Regional Brain Changes in Bipolar I Depression: A Functional Magnetic Resonance Imaging Study. Bipolar Disord..

[42] Atagun M.I., Balaban O.D., Yesilbas D., Keskinkilic C., Evren C. (2012). Effect of Lateralization on Motor and Mental Speed in Bipolar Disorder. Klin. Psikofarmakol. Bülteni-Bull. Clin. Psychopharmacol..

[43] Bilder R.M., Wu H., Bogerts B., Ashtari M., Robinson D., Woerner M., Lieberman J.A., Degreef G. (1999). Cerebral Volume Asymmetries in Schizophrenia and Mood Disorders: A Quantitative Magnetic Resonance Imaging Study. Int. J. Psychophysiol..

[44] Blumberg H.P., Stern E., Martinez D., Ricketts S., De Asis J., White T., Epstein J., McBride P.A., Eidelberg D., Kocsis J.H. (2000). Increased Anterior Cingulate and Caudate Activity in Bipolar Mania. Biol. Psychiatry.

[45] Bruder G.E., Stewart J.W., Towey J.P., Friedman D., Tenke C.E., Voglmaier M.M., Leite P., Cohen P., Quitkin F.M. (1992). Abnormal Cerebral Laterality in Bipolar Depression: Convergence of Behavioral and Brain Event-Related Potential Findings. Biol. Psychiatry.

[46] Bruder G.E., Schnur D.B., Fergeson P., Mukherjee S., Leite P., Sackeim H.A. (1994). Dichotic-Listening Measures of Brain Laterality in Mania. J. Abnorm. Psychol..

[47] Caligiuri M.P., Brown G.G., Meloy M., Eyler L.T., Kindermann S.S., Eberson S., Frank L.R., Lohr J.B. (2004). A Functional Magnetic Resonance Imaging Study of Cortical Asymmetry in Bipolar Disorder. Bipolar Disord..

[48] Coffman J.A., Bornstein R.A., Olson S.C., Schwarzkopf S.B., Nasrallah H.A. (1990). Cognitive Impairment and Cerebral Structure by MRI in Bipolar Disorder. Biol. Psychiatry.

[49] Dewan M.J., Haldipur C.V., Lane E., Donnelly M.P., Boucher M., Major L.F. (1987). Normal Cerebral Asymmetry in Bipolar Patients. Biol. Psychiatry.

[50] Egorov A.Y., Nikolaenko N.N. (1992). Functional Brain Asymmetry and Visuospatial Perception in Mania, Depression, and Psychotropic Medication. Biol. Psychiatry.

[51] Egorov A.Y., Nikolaenko N.N. (1996). P-7 Affective Disorders Are Accompanied by Cerebral Asymmetry Changes. Eur. Neuropsychopharmacol..

[52] Ferro A., Bonivento C., Delvecchio G., Bellani M., Perlini C., Dusi N., Marinelli V., Ruggeri M., Altamura A.C., Crespo-Facorro B. (2017). Longitudinal Investigation of the Parietal Lobe Anatomy in Bipolar Disorder and Its Association with General Functioning. Psychiatry Res. Neuroimaging.

[53] Florio V., Savazzi S., Conca A., Marzi C.A. (2013). Differential Impairment of Interhemispheric Transmission in Bipolar Disease. Exp. Brain Res..

[54] Harmon-Jones E., Abramson L.Y., Nusslock R., Sigelman J.D., Urosevic S., Turonie L.D., Alloy L.B., Fearn M. (2008). Effect of Bipolar Disorder on Left Frontal Cortical Responses to Goals Differing in Valence and Task Difficulty. Biol. Psychiatry.

[55] Ho N.F., Li Z., Ji F., Wang M., Kuswanto C.N., Sum M.Y., Tng H.Y., Sitoh Y.Y., Sim K., Zhou J. (2017). Hemispheric Lateralization Abnormalities of the White Matter Microstructure in Patients with Schizophrenia and Bipolar Disorder. J. Psychiatry Neurosci..

[56] Javadapour A., Malhi G.S., Ivanovski B., Chen X., Wen W., Sachdev P. (2010). Hippocampal Volumes in Adults with Bipolar Disorder. J. Psychiatry Neurosci..

[57] Kaprinis G., Nimatoudis J., Karavatos A., Kandylis D., Kaprinis S. (1995). Functional Brain Organization in Bipolar Affective Patients during Manic Phase and after Recovery: A Digit Dichotic Listening Study. Percept. Mot. Ski..

[58] Kieseppä T., Van Erp T.G.M., Haukka J., Partonen T., Cannon T.D., Poutanen V.-P., Kaprio J., Lönnqvist J. (2003). Reduced Left Hemispheric White Matter Volume in Twins with Bipolar I Disorder. Biol. Psychiatry.

[59] Koller-Schlaud K., Ströhle A., Bärwolf E., Behr J., Rentzsch J. (2020). EEG Frontal Asymmetry and Theta Power in Unipolar and Bipolar Depression. J. Affect. Disord..

[60] Li D., Liu W., Yan T., Cui X., Zhang Z., Wei J., Ma Y., Zhang N., Xiang J., Wang B. (2020). Disrupted Rich Club Organization of Hemispheric White Matter Networks in Bipolar Disorder. Front. Neuroinform..

[61] Li D., Hao J., Hao J., Cui X., Niu Y., Xiang J., Wang B. (2023). Enhanced Dynamic Laterality Based on Functional Subnetworks in Patients with Bipolar Disorder. Brain Sci..

[62] Lohr J.B., Caligiuri M.P. (1995). Motor Asymmetry, a Neurobiologic Abnormality in the Major Psychoses. Psychiatry Res..

[63] Lyon N., Satz P., Fleming K., Green M.F., Bracha H.S. (1992). Left Turning (Swivel) in Manic Patients. Schizophr. Res..

[64] Maller J.J., Anderson R., Thomson R.H., Rosenfeld J.V., Daskalakis Z.J., Fitzgerald P.B. (2015). Occipital Bending (Yakovlevian Torque) in Bipolar Depression. Psychiatry Res. Neuroimaging.

[65] Manelis A., Hu H., Miceli R., Satz S., Lau R., Iyengar S., Swartz H. (2024). The Relationship between the Size and Asymmetry of the Lateral Ventricles and Cortical Myelin Content in Individuals with Mood Disorders 2024. medRxiv.

[66] Najt P., Hausmann M., Weis S. (2013). Altered Resting State Functional Connectivity of the Amygdala in Bipolar Disorder. Functional Cerebral Asymmetries of Emotional Processes in the Healthy and Bipolar Brain.

[67] Najt P., Bayer U., Hausmann M. (2013). Right Fronto-Parietal Dysfunction Underlying Spatial Attention in Bipolar Disorder. Psychiatry Res..

[68] Najt P., Hausmann M. (2014). Atypical Right Hemispheric Functioning in the Euthymic State of Bipolar Affective Disorder. Psychiatry Res..

[69] Nikolaenko N.N., Egorov A.Y., Freiman E.A. (1997). Representation Activity of The Right and Left Hemispheres of the Brain. Behav. Neurol..

[70] Nugent A.C., Milham M.P., Bain E.E., Mah L., Cannon D.M., Marrett S., Zarate C.A., Pine D.S., Price J.L., Drevets W.C. (2006). Cortical Abnormalities in Bipolar Disorder Investigated with MRI and Voxel-Based Morphometry. NeuroImage.

[71] Nusslock R., Harmon-Jones E., Alloy L.B., Urosevic S., Goldstein K., Abramson L.Y. (2012). Elevated Left Mid-Frontal Cortical Activity Prospectively Predicts Conversion to Bipolar I Disorder. J. Abnorm. Psychol..

[72] Padovan G.B. (2018). +3 Psychoses, Language and Brain Asymmetry: fMRI Connectivity Alterations in Bipolar Disorders. Ph.D. Thesis.

[73] Pearlson G.D., Barta P.E., Powers R.E., Menon R.R., Richards S.S., Aylward E.H., Federman E.B., Chase G.A., Petty R.G., Tien A.Y. (1997). Medial and Superior Temporal Gyral Volumes and Cerebral Asymmetry in Schizophrenia versus Bipolar Disorder. Biol. Psychiatry.

[74] Pettigrew J.D., Miller S.M. (1998). A ‘Sticky’ Interhemispheric Switch in Bipolar Disorder?. Proc. R. Soc. Lond. B.

[75] Pinto D., Martins R., Macedo A., Castelo Branco M., Valente Duarte J., Madeira N. (2023). Brain Hemispheric Asymmetry in Schizophrenia and Bipolar Disorder. J. Clin. Med..

[76] Radonić E., Henigsberg N., Rados M., Mimica N., Folnegović-Smalc V. (2008). Temporal Lobe Volume in Disorders with Psychotic Features. Coll. Antropol..

[77] Rao N.P., Arasappa R., Reddy N.N., Venkatasubramanian G., Gangadhar B.N. (2010). Antithetical Asymmetry in Schizophrenia and Bipolar Affective Disorder: A Line Bisection Study. Bipolar Disord..

[78] Reite M., Teale P., Rojas D.C., Reite E., Asherin R., Hernandez O. (2009). MEG Auditory Evoked Fields Suggest Altered Structural/Functional Asymmetry in Primary but Not Secondary Auditory Cortex in Bipolar Disorder. Bipolar Disord..

[79] Romeo Z., Marino M., Angrilli A., Semenzato I., Favaro A., Magnolfi G., Padovan G.B., Mantini D., Spironelli C. (2022). Altered Language Network Lateralization in Euthymic Bipolar Patients: A Pilot Study. Transl. Psychiatry.

[80] Royer C., Delcroix N., Leroux E., Alary M., Razafimandimby A., Brazo P., Delamillieure P., Dollfus S. (2015). Functional and Structural Brain Asymmetries in Patients with Schizophrenia and Bipolar Disorders. Schizophr. Res..

[81] Schindler S., Schmidt L., Stroske M., Storch M., Anwander A., Trampel R., Strauß M., Hegerl U., Geyer S., Schönknecht P. (2019). Hypothalamus Enlargement in Mood Disorders. Acta Psychiatr. Scand..

[82] Spironelli C., Fusina F., Bortolomasi M., Angrilli A. (2021). EEG Frontal Asymmetry in Dysthymia, Major Depressive Disorder and Euthymic Bipolar Disorder. Symmetry.

[83] Stratta P., Daneluzzo E., Mattei P., Casacchia M., Rossi A. (1995). Phasic Asymmetries in Phasic Affective Disorders. Biol. Psychiatry.

[84] Sukno F.M., Kelly B.D., Lane A., Katina S., Rojas M.A., Whelan P.F., Waddington J.L. (2024). Loss of Normal Facial Asymmetry in Schizophrenia and Bipolar Disorder: Implications for Development of Brain Asymmetry in Psychotic Illness. Psychiatry Res..

[85] Tas C., Cebi M., Tan O., Hızlı-Sayar G., Tarhan N., Brown E.C. (2015). EEG Power, Cordance and Coherence Differences between Unipolar and Bipolar Depression. J. Affect. Disord..

[86] Tréhout M., Leroux E., Delcroix N., Dollfus S. (2017). Relationships between Corpus Callosum and Language Lateralization in Patients with Schizophrenia and Bipolar Disorders. Bipolar Disord..

[87] Wang Y., Feng Y., Jia Y., Xie Y., Wang W., Guan Y., Zhong S., Zhu D., Huang L. (2013). Absence of Auditory M100 Source Asymmetry in Schizophrenia and Bipolar Disorder: A MEG Study. PLoS ONE.

[88] Wang Y., Jia Y., Feng Y., Zhong S., Xie Y., Wang W., Guan Y., Zhu D., Huang L. (2014). Overlapping Auditory M100 and M200 Abnormalities in Schizophrenia and Bipolar Disorder: A MEG Study. Schizophr. Res..

[89] Wang B., Li T., Zhou M., Zhao S., Niu Y., Wang X., Yan T., Cao R., Xiang J., Li D. (2018). The Abnormality of Topological Asymmetry in Hemispheric Brain Anatomical Networks in Bipolar Disorder. Front. Neurosci..

[90] Wei S., Geng H., Jiang X., Zhou Q., Chang M., Zhou Y., Xu K., Tang Y., Wang F. (2017). Amygdala-Prefrontal Cortex Resting-State Functional Connectivity Varies with First Depressive or Manic Episode in Bipolar Disorder. Neurosci. Lett..

[91] Yamada S., Takahashi S., Ukai S., Tsuji T., Iwatani J., Tsuda K., Kita A., Sakamoto Y., Yamamoto M., Terada M. (2015). Microstructural Abnormalities in Anterior Callosal Fibers and Their Relationship with Cognitive Function in Major Depressive Disorder and Bipolar Disorder: A Tract-Specific Analysis Study. J. Affect. Disord..

[92] Kim M., Anderson J.M., Heilman K.M. (1997). Search Patterns Using the Line Bisection Test for Neglect. Neurology.

[93] Bhadoria R., Watson D., Danson P., Ferrier I., Mcallister V.I., Moore P.B. (2003). Enlargement of the Third Ventricle in Affective Disorders. Indian. J. Psychiatry.

[94] Hauser P., Matochik J., Altshuler L.L., Denicoff K.D., Conrad A., Li X., Post R.M. (2000). MRI-Based Measurements of Temporal Lobe and Ventricular Structures in Patients with Bipolar I and Bipolar II Disorders. J. Affect. Disord..

[95] Hibar D.P., Westlye L.T., van Erp T.G.M., Rasmussen J., Leonardo C.D., Faskowitz J., Haukvik U.K., Hartberg C.B., Doan N.T., Agartz I. (2016). Subcortical Volumetric Abnormalities in Bipolar Disorder. Mol. Psychiatry.

[96] Strakowski S.M., DelBello M.P., Zimmerman M.E., Getz G.E., Mills N.P., Ret J., Shear P., Adler C.M. (2002). Ventricular and Periventricular Structural Volumes in First- Versus Multiple-Episode Bipolar Disorder. Am. J. Psychiatry.

[97] Zhang F., Peng W., Sweeney J.A., Jia Z., Gong Q. (2018). Brain Structure Alterations in Depression: Psychoradiological Evidence. CNS Neurosci. Ther..

[98] Tian F., Wang X., Long X., Roberts N., Feng C., Yue S., Jia Z. (2021). The Correlation of Reduced Fractional Anisotropy in the Cingulum with Suicide Risk in Bipolar Disorder. Front. Psychiatry.

[99] Vederine F.-E., Wessa M., Leboyer M., Houenou J. (2011). A Meta-Analysis of Whole-Brain Diffusion Tensor Imaging Studies in Bipolar Disorder. Prog. Neuro-Psychopharmacol. Biol. Psychiatry.

[100] Versace A., Almeida J.R.C., Hassel S., Walsh N.D., Novelli M., Klein C.R., Kupfer D.J., Phillips M.L. (2008). Elevated Left and Reduced Right Orbitomedial Prefrontal Fractional Anisotropy in Adults with Bipolar Disorder Revealed by Tract-Based Spatial Statistics. Arch. Gen. Psychiatry.

[101] Foley S.F., Bracher-Smith M., Tansey K.E., Harrison J.R., Parker G.D., Caseras X. (2018). Fractional Anisotropy of the Uncinate Fasciculus and Cingulum in Bipolar Disorder Type I, Type II, Unaffected Siblings and Healthy Controls. Br. J. Psychiatry.

[102] Lu L.H., Zhou X.J., Keedy S.K., Reilly J.L., Sweeney J.A. (2011). White Matter Microstructure in Untreated First Episode Bipolar Disorder with Psychosis: Comparison with Schizophrenia. Bipolar Disord..

[103] Macritchie K.A.N., Lloyd A.J., Bastin M.E., Vasudev K., Gallagher P., Eyre R., Marshall I., Wardlaw J.M., Ferrier I.N., Moore P.B. (2010). White Matter Microstructural Abnormalities in Euthymic Bipolar Disorder. Br. J. Psychiatry.

[104] Thiel K., Meinert S., Winter A., Lemke H., Waltemate L., Breuer F., Gruber M., Leenings R., Wüste L., Rüb K. (2023). Reduced Fractional Anisotropy in Bipolar Disorder *v.* Major Depressive Disorder Independent of Current Symptoms. Psychol. Med..

[105] Barysheva M., Jahanshad N., Foland-Ross L., Altshuler L.L., Thompson P.M. (2013). White Matter Microstructural Abnormalities in Bipolar Disorder: A Whole Brain Diffusion Tensor Imaging Study. NeuroImage Clin..

[106] Benedetti F., Yeh P.-H., Bellani M., Radaelli D., Nicoletti M.A., Poletti S., Falini A., Dallaspezia S., Colombo C., Scotti G. (2011). Disruption of White Matter Integrity in Bipolar Depression as a Possible Structural Marker of Illness. Biol. Psychiatry.

[107] Bruno S., Cercignani M., Ron M.A. (2008). White Matter Abnormalities in Bipolar Disorder: A Voxel-based Diffusion Tensor Imaging Study. Bipolar Disord..

[108] Canales-Rodríguez E.J., Pomarol-Clotet E., Radua J., Sarró S., Alonso-Lana S., Del Mar Bonnín C., Goikolea J.M., Maristany T., García-Álvarez R., Vieta E. (2014). Structural Abnormalities in Bipolar Euthymia: A Multicontrast Molecular Diffusion Imaging Study. Biol. Psychiatry.

[109] Heng S., Song A.W., Sim K. (2010). White Matter Abnormalities in Bipolar Disorder: Insights from Diffusion Tensor Imaging Studies. J. Neural Transm..

[110] Zanetti M.V., Jackowski M.P., Versace A., Almeida J.R.C., Hassel S., Duran F.L.S., Busatto G.F., Kupfer D.J., Phillips M.L. (2009). State-Dependent Microstructural White Matter Changes in Bipolar I Depression. Eur. Arch. Psychiatry Clin. Neurosci..

[111] Dwyer J.H., Rinn W.E. (1981). The Role of the Right Hemisphere in Contextual Inference. Neuropsychologia.

[112] Gainotti G. (2011). What the Study of Voice Recognition in Normal Subjects and Brain-Damaged Patients Tells Us about Models of Familiar People Recognition. Neuropsychologia.

[113] Barlattani T., Cavatassi A., Bologna A., Socci V., Trebbi E., Malavolta M., Rossi A., Martiadis V., Tomasetti C., De Berardis D. (2025). Glymphatic system and psychiatric disorders: Need for a new paradigm?. Front. Psychiatry.

[114] Chepenik L.G., Wang F., Spencer L., Spann M., Kalmar J.H., Womer F., Kale Edmiston E., Pittman B., Blumberg H.P. (2012). Structure–Function Associations in Hippocampus in Bipolar Disorder. Biol. Psychol..

[115] Otten M., Meeter M. (2015). Hippocampal Structure and Function in Individuals with Bipolar Disorder: A Systematic Review. J. Affect. Disord..

[116] Ward L.M. (2013). The Thalamus: Gateway to the Mind. WIRES Cogn. Sci..

[117] Gouveia F.V., Hamani C., Fonoff E.T., Brentani H., Alho E.J.L., De Morais R.M.C.B., De Souza A.L., Rigonatti S.P., Martinez R.C.R. (2019). Amygdala and Hypothalamus: Historical Overview with Focus on Aggression. Neurosurgery.

[118] Gründemann J., Bitterman Y., Lu T., Krabbe S., Grewe B.F., Schnitzer M.J., Lüthi A. (2019). Amygdala Ensembles Encode Behavioral States. Science.

[119] Hoang I.B., Sharpe M.J. (2021). The Basolateral Amygdala and Lateral Hypothalamus Bias Learning towards Motivationally Significant Events. Curr. Opin. Behav. Sci..

[120] Cecil K.M., DelBello M.P., Morey R., Strakowski S.M. (2002). Frontal Lobe Differences in Bipolar Disorder as Determined by Proton MR Spectroscopy. Bipolar Disord..

[121] Haldane M., Frangou S. (2005). Maudsley Bipolar Disorder Project: Insights Sobre o Papel Do Córtex Pré-Frontal Em Pacientes Com Transtorno de Humor Bipolar Tipo I. Rev. Psiquiatr. Rio Gd. Sul.

[122] Hibar D.P., Westlye L.T., Doan N.T., Jahanshad N., Cheung J.W., Ching C.R.K., Versace A., Bilderbeck A.C., Uhlmann A., Mwangi B. (2018). Cortical Abnormalities in Bipolar Disorder: An MRI Analysis of 6503 Individuals from the ENIGMA Bipolar Disorder Working Group. Mol. Psychiatry.

[123] López-Larson M.P., DelBello M.P., Zimmerman M.E., Schwiers M.L., Strakowski S.M. (2002). Regional Prefrontal Gray and White Matter Abnormalities in Bipolar Disorder. Biol. Psychiatry.

[124] Lyoo I.K., Sung Y.H., Dager S.R., Friedman S.D., Lee J., Kim S.J., Kim N., Dunner D.L., Renshaw P.F. (2006). Regional Cerebral Cortical Thinning in Bipolar Disorder. Bipolar Disord..

[125] Macoveanu J., Freeman K.O., Kjærstad H.L., Knudsen G.M., Kessing L.V., Miskowiak K.W. (2021). Structural Brain Abnormalities Associated with Cognitive Impairments in Bipolar Disorder. Acta Psychiatr. Scand..

[126] Stanfield A.C., Moorhead T.W.J., Job D.E., McKirdy J., Sussmann J.E., Hall J., Giles S., Johnstone E.C., Lawrie S.M., McIntosh A.M. (2009). Structural Abnormalities of Ventrolateral and Orbitofrontal Cortex in Patients with Familial Bipolar Disorder. Bipolar Disord..

[127] Rolls E.T., Grabenhorst F. (2008). The Orbitofrontal Cortex and beyond: From Affect to Decision-Making. Prog. Neurobiol..

[128] Hartikainen K.M. (2021). Emotion-Attention Interaction in the Right Hemisphere. Brain Sci..

[129] McCrea S. (2008). Bipolar Disorder and Neurophysiologic Mechanisms. Neuropsychiatr. Dis. Treat..

[130] Wang B., Yang L., Yan W., An W., Xiang J., Li D. (2023). Brain Asymmetry: A Novel Perspective on Hemispheric Network. Brain Sci. Adv..

[131] Cardinale R.C., Shih P., Fishman I., Ford L.M., Müller R.-A. (2013). Pervasive Rightward Asymmetry Shifts of Functional Networks in Autism Spectrum Disorder. JAMA Psychiatry.

[132] Floris D.L., Lai M., Auer T., Lombardo M.V., Ecker C., Chakrabarti B., Wheelwright S.J., Bullmore E.T., Murphy D.G.M., Baron-Cohen S. (2016). Atypically Rightward Cerebral Asymmetry in Male Adults with Autism Stratifies Individuals with and without Language Delay. Hum. Brain Mapp..

[133] Geng S., Dai Y., Rolls E.T., Liu Y., Zhang Y., Deng L., Chen Z., Feng J., Li F., Cao M. (2025). Rightward Brain Structural Asymmetry in Young Children with Autism. Mol. Psychiatry.

[134] Miyata J., Sasamoto A., Koelkebeck K., Hirao K., Ueda K., Kawada R., Fujimoto S., Tanaka Y., Kubota M., Fukuyama H. (2012). Abnormal Asymmetry of White Matter Integrity in Schizophrenia Revealed by Voxelwise Diffusion Tensor Imaging. Hum. Brain Mapp..

[135] Sallet P.C., Elkis H., Alves T.M., Oliveira J.R., Sassi E., De Castro C.C., Busatto G.F., Gattaz W.F. (2003). Reduced Cortical Folding in Schizophrenia: An MRI Morphometric Study. Am. J. Psychiatry.

[136] Davidson R.J. (1992). Anterior Cerebral Asymmetry and the Nature of Emotion. Brain Cogn..

[137] Hecht D. (2010). Depression and the Hyperactive Right-Hemisphere. Neurosci. Res..

[138] Davidson R.J. (1984). Affect, Cognition, and Hemispheric Specialization. Emotions, Cognition, and Behavior.

[139] Cotovio G., Oliveira-Maia A.J. (2022). Functional Neuroanatomy of Mania. Transl. Psychiatry.

[140] Liotti M., Tucker D.M. (1992). Right Hemisphere Sensitivity to Arousal and Depression. Brain Cogn..

[141] Otto M.W., Yeo R.A., Dougher M.J. (1987). Right Hemisphere Involvement in Depression: Toward a Neuropsychological Theory of Negative Affective Experiences. Biol. Psychiatry.

[142] Christianson S.A., Saisa J., Garvill J., Silfvenius H. (1993). Hemisphere Inactivation and Mood-State Changes. Brain Cogn..

[143] Heilman K.M., Bowers D., Valenstein E., Watson R.T. (1986). The Right Hemisphere: Neuropsychological Functions. J. Neurosurg..

[144] Ross E.D. (1984). Right Hemisphere’s Role in Language, Affective Behavior and Emotion. Trends Neurosci..

[145] Wedding D., Stalans L. (1985). Hemispheric Differences in the Perception of Positive and Negative Faces. Int. J. Neurosci..

